# Supplementary material for: Using the consolidated framework for implementation research to evaluate a model of community-engaged research in advance care planning
Source: PLoS One. 2026 Mar 27;21(3):e0343235. doi: 10.1371/journal.pone.0343235 (PMC13028479; doi:10.1371/journal.pone.0343235)
Supplement: Attachment — Submitted filename: PLOS One Response to Reviewer Table_FINAL_9.16.25.docx [file pone.0343235.s002.docx]

| Comment | Revision |
| --- | --- |
| 1. Please ensure that your manuscript meets PLOS ONE's style requirements, including those for file naming. |  |
| “The authors declare that the research was conducted in the absence of any commercial or financial relationships that could be construed as a potential conflict of interest. Dr. Van Scoy is an unpaid scientific advisor for Common Practice, LLC.”  Please confirm that this does not alter your adherence to all PLOS ONE policies on sharing data and materials, by including the following statement: "This does not alter our adherence to  PLOS ONE policies on sharing data and materials.” (as detailed online in our guide for authors [http://journals.plos.org/plosone/s/competing-interests](https://urldefense.com/v3/__http:/journals.plos.org/plosone/s/competing-interests__;!!Ls64Rlj6!yZiyntKQWlPeBAkuWF5gecwtThv4GF5R3edHmUFYsRIqpTMQDI9WgTIVihz8tJnCLEGIHhzLMJrMMOHH9NIw3EChYl0$)).  If there are restrictions on sharing of data and/or materials, please state these. Please note that we cannot proceed with consideration of your article until this information has been declared.  Please include your updated Competing Interests statement in your cover letter; we will change the online submission form on your behalf. | Thank you for this comment. Our updated Competing Interests statement has been included with the updated cover letter for this resubmission. We have also disclosed two other financial relationships that are not a conflict of interest, but the authors would like to share in the spirit of transparency. |
| 3. One of the noted authors is a group or consortium: The Project Talk Trial Collaborative Working Group Members  In addition to naming the author group, please list the individual authors and affiliations within this group in the acknowledgments section of your manuscript. Please also indicate clearly a lead author for this group along with a contact email address. | Affiliations and the lead author has been added to the acknowledgement section |
| 4. Please ensure that you refer to Figure 1 in your text as, if accepted, production will need this reference to link the reader to the figure. | Thank you for this comment, after consideration, the authors have determined the figure does not support the reader’s understanding of the study, so the authors have made the decision to remove the figure from the manuscript. |
| 5. We note that Figures 1 and 2 in your submission contain map images which may be copyrighted. All PLOS content is published under the Creative Commons Attribution License (CC BY 4.0), which means that the manuscript, images, and Supporting Information files will be freely available online, and any third party is permitted to access, download, copy, distribute, and use these materials in any way, even commercially, with proper attribution. For these reasons, we cannot publish previously copyrighted maps or satellite images created using proprietary data, such as Google software (Google Maps, Street View, and Earth). For more information, see our copyright guidelines: [http://journals.plos.org/plosone/s/licenses-and-copyright](https://urldefense.com/v3/__http:/journals.plos.org/plosone/s/licenses-and-copyright__;!!Ls64Rlj6!yZiyntKQWlPeBAkuWF5gecwtThv4GF5R3edHmUFYsRIqpTMQDI9WgTIVihz8tJnCLEGIHhzLMJrMMOHH9NIwDoFz6c4$).   We require you to either (1) present written permission from the copyright holder to publish these figures specifically under the CC BY 4.0 license, or (2) remove the figures from your submission:   1) You may seek permission from the original copyright holder of Figures 1 and 2 to publish the content specifically under the CC BY 4.0 license.     We recommend that you contact the original copyright holder with the Content Permission Form ([http://journals.plos.org/plosone/s/file?id=7c09/content-permission-form.pdf](https://urldefense.com/v3/__http:/journals.plos.org/plosone/s/file?id=7c09*content-permission-form.pdf__;Lw!!Ls64Rlj6!yZiyntKQWlPeBAkuWF5gecwtThv4GF5R3edHmUFYsRIqpTMQDI9WgTIVihz8tJnCLEGIHhzLMJrMMOHH9NIwe9YSAuE$)) and the following text: “I request permission for the open-access journal PLOS ONE to publish XXX under the Creative Commons Attribution License (CCAL) CC BY 4.0 ([http://creativecommons.org/licenses/by/4.0/](https://urldefense.com/v3/__http:/creativecommons.org/licenses/by/4.0/__;!!Ls64Rlj6!yZiyntKQWlPeBAkuWF5gecwtThv4GF5R3edHmUFYsRIqpTMQDI9WgTIVihz8tJnCLEGIHhzLMJrMMOHH9NIwueNs0VE$)). Please be aware that this license allows unrestricted use and distribution, even commercially, by third parties. Please reply and provide explicit written permission to publish XXX under a CC BY license and complete the attached form.”   Please upload the completed Content Permission Form or other proof of granted permissions as an ""Other"" file with your submission.   In the figure caption of the copyrighted figure, please include the following text: “Reprinted from [ref] under a CC BY license, with permission from [name of publisher], original copyright [original copyright year].” | We would like to clarify that Figures 1 and 2 in our manuscript do not contain copyrighted material.  Figures 1 and 2 were created entirely by the authors. No proprietary mapping services such as Google Maps, Google Earth, or other commercial mapping platforms were used in the creation of these figures. As the original creators of these maps, we retain full copyright and are able to publish them under the CC BY 4.0 license without any restrictions. Figure 1 will no longer be used in the manuscript. |
| 7. We note that there is identifying data in the Supporting Information file < UTF-8PTT approvals.pdf>. Due to the inclusion of these potentially identifying data, we have removed this file from your file inventory. Prior to sharing human research participant data, authors should consult with an ethics committee to ensure data are shared in accordance with participant consent and all applicable local laws.  Data sharing should never compromise participant privacy. It is therefore not appropriate to publicly share personally identifiable data on human research participants. The following are examples of data that should not be shared:  -Name, initials, physical address -Ages more specific than whole numbers -Internet protocol (IP) address -Specific dates (birth dates, death dates, examination dates, etc.) -Contact information such as phone number or email address -Location data -ID numbers that seem specific (long numbers, include initials, titled “Hospital ID”) rather than random (small numbers in numerical order)  Data that are not directly identifying may also be inappropriate to share, as in combination they can become identifying. For example, data collected from a small group of participants, vulnerable populations, or private groups should not be shared if they involve indirect identifiers (such as sex, ethnicity, location, etc.) that may risk the identification of study participants.  Additional guidance on preparing raw data for publication can be found in our Data Policy ([https://journals.plos.org/plosone/s/data-availability#loc-human-research-participant-data-and-other-sensitive-data](https://urldefense.com/v3/__https:/journals.plos.org/plosone/s/data-availability*loc-human-research-participant-data-and-other-sensitive-data__;Iw!!Ls64Rlj6!yZiyntKQWlPeBAkuWF5gecwtThv4GF5R3edHmUFYsRIqpTMQDI9WgTIVihz8tJnCLEGIHhzLMJrMMOHH9NIwRYIG76s$)) and in the following article: [http://www.bmj.com/content/340/bmj.c181.long](https://urldefense.com/v3/__http:/www.bmj.com/content/340/bmj.c181.long__;!!Ls64Rlj6!yZiyntKQWlPeBAkuWF5gecwtThv4GF5R3edHmUFYsRIqpTMQDI9WgTIVihz8tJnCLEGIHhzLMJrMMOHH9NIwv4sI7PE$).  Please remove or anonymize all personal information ensure that the data shared are in accordance with participant consent, and re-upload a fully anonymized data set. Please note that spreadsheet columns with personal information must be removed and not hidden as all hidden columns will appear in the published file. | We appreciate the reviewer's attention to data privacy concerns. However, we believe there may be a misunderstanding regarding the nature and content of the "UTF-8PTT approvals.pdf" file that was removed from our submission.  The UTF-8PTT approvals.pdf file does not contain any participant data, identifying or otherwise. This document exclusively contains the names and signatures of our Institutional Review Board (IRB) analysts and committee members who approved various protocol modifications throughout the study period. No participant information, demographic data, or any other human subjects data appears in this file.  At initial manuscript submission, we were requested to provide documentation demonstrating continuous IRB oversight throughout our research period. Since our IRB does not conduct continuing review for this particular study category, we compiled a comprehensive document showing our modification approval history from initial submission to present. This document was intended to demonstrate ongoing ethical oversight and compliance,.  Given that our submitted documentation has been deemed unacceptable, we respectfully request specific guidance on how we should demonstrate continuous IRB oversight for this study. We are committed to meeting PLOS ONE's requirements while maintaining appropriate confidentiality of institutional personnel information. |
| Reviewer #1: Introduction: 1. In the second paragraph, the author has used stated 'burdensome and unwanted treatments', I believe this wordings is conflicting and would suggest authors to reword the statement. | Thank you for this comment. “Burdensome and unwanted” has been removed from this sentence. |
| 1. Under CFIR mapping, two authors reviewed the transcripts. What was the procedure followed in instances of difference of opinion? | Thank you for this helpful comment. We have clarified our analytic process in the manuscript. Specifically, we now state that two authors independently coded transcripts, discussed any disagreements to reach consensus, and when consensus could not be reached, the implementation science faculty lead of the study (WC) adjudicated the final decision. We believe this addition improves clarity and transparency of our methods. |
| 2. The author conducted 24 interviews. Why only 24? Is there a reasoning behind sample size fixed at 24 host participants? | Thank you for this comment. We now include details of qualitative saturation which was what informed our decision to begin analysis of interviews after n=26 (see comment below). We have been included in the “Qualitatiave Interviews” section to better address why 26 interviews were included in this sample. |
| 3. Were there any non English audio files? If yes, how were they translated and back translated before transcribing? | Thank you for this comment. All interviews were conducted in English, and this has been reflected in the “Qualitatiave Interviews” section. |
| 4. Any particular reason why both analyst didn't code similar number of transcripts? | Thank you for this comment. The qualitative analysis of the host interviews was conducted as part of dissertation research led by the first author (EV). After calibrating coding on 20% of the data, coder EV proceeded to code the majority of the interviews, as this work constituted her dissertation research and the second coder was unable to take on additional files. |
| 1. Throughout the methodology, the authors have stated 24 host participants. However, under host characteristics section, the manuscripts reads as 26. Kindly clarify. | Thank you for catching this error. The host interview sample included n=26 interviews, and this has been updated in the methodology section. |
| 2. Same in table 1. | Thank you for this comment and apologies for any confusion. Table 1 is correct with n=26. |
| Firstly, the rationale for selecting CFIR over other frameworks (such as RE-AIM or the Theoretical Domains Framework) needs to be more explicitly stated. While CFIR is comprehensive, its application to community-engaged research—especially in culturally sensitive domains like ACP—requires adaptation and justification, particularly for constructs that may not fully resonate with community dynamics. | CFIR is meta-theoretical, it includes constructs from 19 frameworks or related theories. In particular, CFIR covers the RE-AIM through the inclusion of PRISM domains. Although CFIR did not explicitly mention TDF, many of its domains and constructs are integrated into CFIR. Note that TDF identifies influences on professional behaviors related to implementation of best practices; this is covered under the 1) characteristics of individuals and 2) process domains in CFIR.  With regard to the application of CFIR to this community-engaged research, we agree that the CFIR has most often been used within healthcare settings but has also been used across a range of community research studies. For these reasons we selected CFIR to guide the data collection and analysis of the present study. |
| The manuscript could benefit from a more detailed discussion on how CFIR constructs were operationalized during data collection and analysis. For example, were interview guides or coding frameworks co-developed with community partners? Such steps would support the authenticity of community engagement and demonstrate methodological rigor. Additionally, the process for ensuring inter-coder reliability when mapping qualitative data to CFIR constructs is unclear. More transparency here would bolster confidence in the study’s analytical rigor. | We thank the reviewer for this helpful suggestion. The interview guide was co-developed with members of the trial’s collaborative working group, which included both community and academic partners. The guide was presented to the group, reviewed, and revised based on their feedback, with the final version approved by the group. In addition, an implementation science expert (WC) reviewed the mapping of data to CFIR constructs. These details have been added to the revised manuscript. |
| In terms of findings, the manuscript thoughtfully identifies relevant CFIR domains such as “Inner Setting” and “Characteristics of Individuals,” but it is less clear how these domains interacted or evolved over time within the implementation process. A visual depiction or matrix linking specific constructs to key findings would enhance clarity and utility for readers and future researchers. | We appreciate the reviewer’s thoughtful suggestion. While we agree that a visual depiction can be useful, in this case we felt that a figure or matrix would oversimplify the nuanced ways in which CFIR domains interacted and evolved over time. Because our goal was to capture the complexity and contextual richness of the implementation process, we chose to present these dynamics narratively, which allows for greater depth and nuance than a static visual could convey. This approach is consistent with established qualitative reporting standards (e.g., COREQ), which emphasize detailed description and contextualization over reduction to schematic forms. |
| The manuscript also briefly touches on adaptability and sustainability—two crucial components of implementation success—but does not delve deeply into how these were influenced by the broader socio-political context or varying community characteristics. Exploring these nuances could offer richer insight into transferability, especially across underserved or diverse populations. | Our data did not include exploration of sociopolitical context and since the political landscape has changed so dramatically since this data was collected, we believe such a discussion is out of the scope of our manuscript. We have added discussion about how sociocultural differences have been seen in other studies resulting from this work (Costigan et. al). |
| A strength of the manuscript is its integration of community voices throughout the evaluation process. However, it would be helpful to understand whether community stakeholders were involved in selecting or interpreting CFIR constructs, and how their perspectives were integrated into the final framework application. This would further demonstrate the authenticity of the “community-engaged” approach. | Thank you for this comment, we have added how our trial advisory committee reviewed and provided feedback on the interview guide. |
| From an ethics and publication standpoint, there are no immediate concerns regarding dual publication or research misconduct. However, the authors should clarify if this model has been described in any prior evaluations or pilot studies. If so, appropriate cross-referencing or citation should be provided to ensure transparency and avoid redundancy. | Thank you for this thoughtful comment. This model has not been published elsewhere, and this clarification has been added to the “Key Structural Elements” section. |
| In the methods section, under Qualitative Analysis, please include the company name and country of origin after mentioning MAXQDA (v. 24). For example: MAXQDA (v. 24; VERBI GmbH, Berlin, Germany). | Thank you for this comment. Additional information on MAXQDA has been added. |
| In the main text, revise “Black communities (39%)” to “Black communities (38.5%)” to match Table 1. | Thank you for this comment. This revision has been made. |
| Revise the regional distribution text to reflect one decimal place, consistent with Table 1 formatting: “Hosts from a total of 18 states were included in the sample from across the Northeast (19.2%), Midwest (15.4%), South (42.3%), and West (23.1%).” | Thank you for this comment. This revision has been made. |
| Update the “Urban Black” entry from 38.4% to 38.5%. Table 1 | Thank you for this comment. This revision has been made. |
| Revise “West” from 23.2% to 23.1%. Table 1 | Thank you for this comment. This revision has been made. |
| Figure 1 Quality: The image quality of Figure 1 is currently suboptimal. Please consider uploading a higher-resolution version to improve clarity for readers. | Thank you for this comment, as described above, we have chosen to not include Figure 1 in the manuscript. |
